# The *XChemExplorer* graphical workflow tool for routine or large-scale protein–ligand structure determination

**DOI:** 10.1107/S2059798316020234

**Published:** 2017-02-24

**Authors:** Tobias Krojer, Romain Talon, Nicholas Pearce, Patrick Collins, Alice Douangamath, Jose Brandao-Neto, Alexandre Dias, Brian Marsden, Frank von Delft

**Affiliations:** a Structural Genomics Consortium, University of Oxford, Roosevelt Drive, Oxford OX3 7DQ, England; b Diamond Light Source Ltd, Harwell Science and Innovation Campus, Didcot OX11 0QX, England; c Kennedy Institute of Rheumatology, University of Oxford, Roosevelt Drive, Oxford OX3 7FY, England; d Department of Biochemistry, University of Johannesburg, Auckland Park 2006, South Africa

**Keywords:** *XChemExplorer*, *PanDDA*, structure-based ligand design, protein–ligand structure, fragment screening

## Abstract

*XChemExplorer* (*XCE*) is a data-management and workflow tool to support large-scale simultaneous analysis of protein–ligand complexes during structure-based ligand discovery (SBLD). The user interfaces of established crystallo­graphic software packages such as *CCP*4 [Winn *et al.* (2011 ▸), *Acta Cryst.* D**67**, 235–242] or *PHENIX* [Adams *et al.* (2010 ▸), *Acta Cryst.* D**66**, 213–221] have entrenched the paradigm that a ‘project’ is concerned with solving one structure. This does not hold for SBLD, where many almost identical structures need to be solved and analysed quickly in one batch of work. Functionality to track progress and annotate structures is essential. *XCE* provides an intuitive graphical user interface which guides the user from data processing, initial map calculation, ligand identification and refinement up until data dissemination. It provides multiple entry points depending on the need of each project, enables batch processing of multiple data sets and records metadata, progress and annotations in an SQLite database. *XCE* is freely available and works on any Linux and Mac OS X system, and the only dependency is to have the latest version of *CCP*4 installed. The design and usage of this tool are described here, and its usefulness is demonstrated in the context of fragment-screening campaigns at the Diamond Light Source. It is routinely used to analyse projects comprising 1000 data sets or more, and therefore scales well to even very large ligand-design projects.

## Abstract 2

*XChemExplorer* is a graphical workflow and data-management tool for the parallel determination of protein–ligand complexes. Its implementation, usage and application are described here.

## 1. Introduction   

Protein crystallography is a cornerstone of structure-based ligand design (SBLD; Blundell *et al.*, 2002 ▸), providing the molecular details of how newly synthesized compounds interact with the protein of interest in order to drive the compound-design process. This requires iterative cycles of design and synthesis of compounds with improved binding characteristics, based on structural information of previous protein–ligand complexes and biophysical measurements (Carvalho *et al.*, 2009 ▸). In the past, SBLD was the preserve of industry, but academic groups have lately started to pick up on this approach (Hole *et al.*, 2013 ▸; Brem *et al.*, 2016 ▸).

Depending on the magnitude of the project and its resources, every development cycle may yield tens of novel compounds, each of which requires a protein–ligand structure, either by co-crystallization or soaking into preformed crystals. The widespread availability of third-generation synchrotron sources which are equipped with reliable robotic sample changers and fast X-ray detectors has made it possible to collect hundreds of data sets during a single visit (Broennimann *et al.*, 2006 ▸; Nurizzo *et al.*, 2016 ▸). The extreme case is crystallographic fragment screening, in which hundreds of similar data sets are collected rapidly and automatically (Bauman *et al.*, 2013 ▸; Schiebel *et al.*, 2016 ▸). In 2012, Wasserman and coworkers estimated that industry determines in excess of 10 000 protein–ligand structures per year (Wasserman *et al.*, 2012 ▸), and in the light of ever-improving hardware and software this number is likely to increase manyfold in the near future. For instance, the Oxford site of the Structural Genomics Consortium (SGC) alone collected 960 data sets from different protein–ligand complexes in 2015.

The success and throughput of SBLD projects depends on the availability of robust and well diffracting crystal systems (Danley, 2006 ▸). Hence, from a crystallographic point of view, data collection, processing and refinement of such samples might appear to be algorithmically trivial: most crystals will be nearly isomorphous, so that phasing is achieved by simple molecular substitution, while adjustments to the model are usually restricted to the region around the respective ligand-binding site.

Crystallographic software packages have undergone a remarkable degree of automation over the last decade which facilitates all steps of structure determination, starting with data processing (Vonrhein *et al.*, 2011 ▸; Winter, 2010 ▸; Sparta *et al.*, 2016 ▸; Grochulski *et al.*, 2012 ▸), through phasing (Holton & Alber, 2004 ▸; Keegan & Winn, 2008 ▸; Ness *et al.*, 2004 ▸; Panjikar *et al.*, 2005 ▸; Terwilliger *et al.*, 2009 ▸) and model building (Cowtan, 2006 ▸; Porebski *et al.*, 2016 ▸; Terwilliger *et al.*, 2008 ▸), all the way to refinement (Joosten *et al.*, 2012 ▸). Additionally, pipelines have become available to specifically support the solution of protein–ligand structures (Echols *et al.*, 2014 ▸).

However, what remains unaddressed is the logistics of analysing large numbers of data sets. Crystallographic software suites such as *CCP*4 or *PHENIX* have not evolved beyond the early paradigm that one project leads to one structure, a design that is adequate for the determination of novel or otherwise challenging crystal structures but does not really reflect the requirements of SBLD projects. In SBLD, a single project consists of multiple structures, all of which need to be analysed in relation to each other. Additionally, protein–ligand structure solution is usually a linear, well defined process and mostly requires the repetitive usage of a limited number of software tools. This seemingly simple workflow becomes tedious because it requires the user to relaunch the same processes over and over again. Ideally, users should be able to analyse, build and refine multiple structures within one software instance without having to open and close different programs.

The *AutoSolve* platform from Astex (Mooij *et al.*, 2006 ▸) represents one solution to the problem, and it can be assumed that many industrial or industry-like groups have developed similar software solutions that tie in with their internal databases. However, these solutions are not publicly available and, even if they were, such systems would not be trivial to set up or to maintain. There exist no up-to-date workflow-management tools to support multi-data-set analysis that are available to the broad crystallographic community.

Here, we present *XChemExplorer* (*XCE*), a graphical, data-management and workflow tool that brings together established programs that are typically used to process, solve and refine protein–ligand structures. *XCE* does not try to automate the structure-determination process, but it serves as the control centre for running the necessary programs and allows the user to annotate each experiment easily but thoroughly. *XCE* was originally developed to support crystallographic fragment screening at the Diamond Light Source, but it is a generic tool that can be used for any project that needs to solve many similar protein–ligand structures.

## 2. Philosophy   

**Figure 1:**
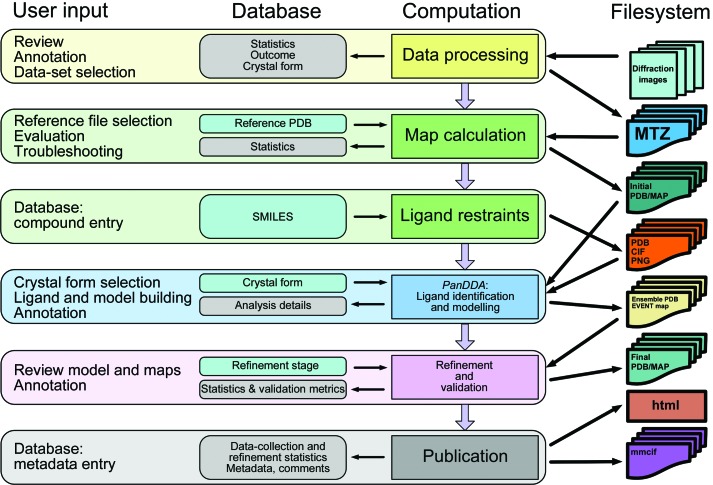
Conceptual design of *XChemExplorer*. The diagram illustrates the supported workflow and the interplay of database and filesystem. User input is mediated through the graphical user interface.

**Figure 2:**
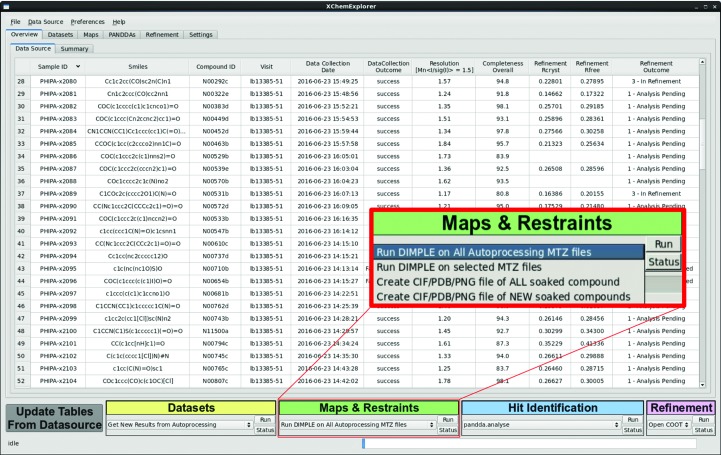
Overview and design principle of the *XChemExplorer* GUI. The Overview tab shows the contents of the database. The general design principle is the same for all tabs: information and parameters which are specific for each milestone during structure solution are displayed in the centre. The coloured action boxes at the bottom of the window bundle a set of commands that are relevant for each stage of the structure-determination process. The inset gives an example of the different processes that can be executed in the Maps & Restraints tab. The grey button at the bottom left corner is for refreshing the tables of the GUI. Status messages and a progress bar in the lowermost part of the window provide feedback and inform about ongoing processes.

**Figure 3:**
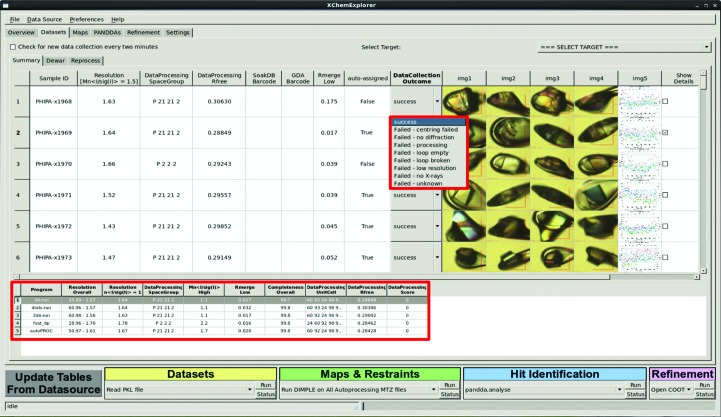
The Datasets panel which allows users to review data collected at the Diamond Light Source. Selected data-collection statistics, crystal images after alignment and a drop-down widget for annotating the outcome of the experiment are displayed for each crystal. The upper red box shows a selection of the various data-collection outcome annotations. The Show Details checkbox is for displaying an extended view of the output from all auto-processing pipelines; the lower red box shows detailed data-collection statistics for different auto-processing pipelines. It is possible to manually choose any outcome from the respective table.

**Figure 4:**
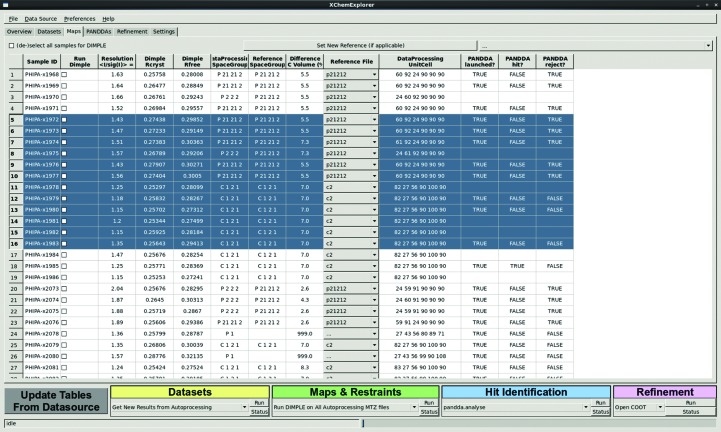
The Maps & Restraints panel which allows users to calculate initial maps and generate ligand coordinates and restraints. The central table gives an overview of all data sets that can be used for initial map calculation with *DIMPLE*. *R*_cryst_ and *R*_free_ are displayed if initial refinement has already taken place. Like all other tables, the table can be sorted by any of the displayed columns. *XCE* tries to automatically assign a suitable reference file for input into *DIMPLE*. The drop-down menu displays the currently selected reference file, but it is possible to select any other file from the list. The last three columns indicate the status of each data set, *i.e.* whether they were already subject to analysis and inspection with the *PanDDA* algorithm. Ranges of rows can be selected and marked for map calculation with *DIMPLE*.

**Figure 5:**
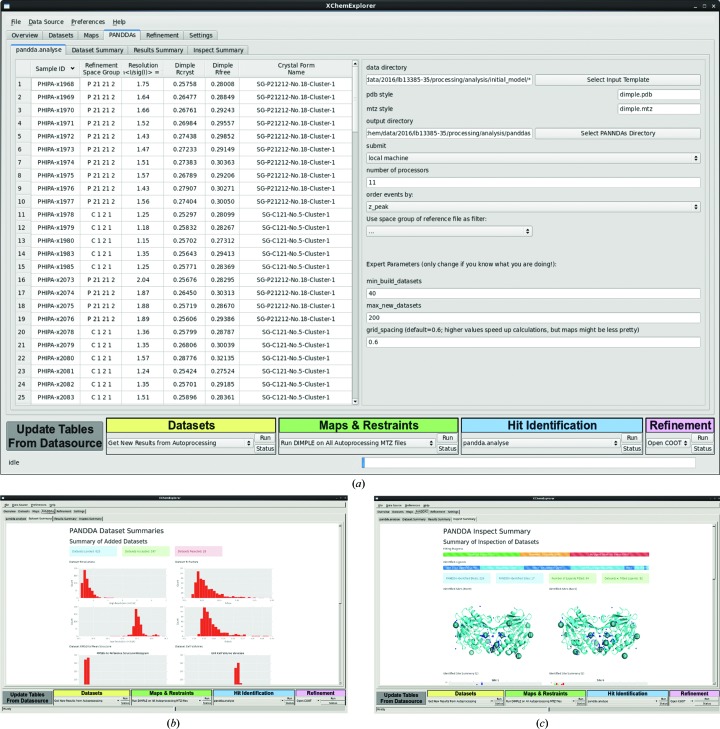
The PanDDA panel allows the configuration of calculations by the *PanDDA* software. (*a*) The left table provides an overview of all the data sets that are available for input into *PanDDA*; the right side displays the currently selected input parameters for *pandda.analyse*. These are relevant mostly when multiple crystal forms are available. (*b*) *PanDDA* data-set summary. (*c*) *PanDDA* inspection summary.

Protein–ligand structure determination may be an algorithmically simple and linear process, but it turns into a challenge when many samples are analysed as part of an SBLD project which can go on for months and years. Not only are there specific demands on project design, but there have to be mechanisms to capture progress and results. This becomes exponentially challenging as the numbers of data sets become large, unless it is explicitly supported by software. For example, compounds often do not bind to a protein and the reason for this is not always immediately clear. While the experiment ultimately remains unsuccessful, the reason for the failure may be unrelated to the compound, and become evident only when every step of that particular experiment is analysed. *XCE* records compound information, metadata, file locations, observations, outcome and status of the project in a database. It serves as a funnel which channels disparate data into a grid so that it becomes comprehensible for the scientist. The program does not try to make decisions for the crystallo­grapher, but it provides the tools to evaluate each experiment, assign a result and (re)run calculations in batches. The recommended workflow (Fig. 1 ▸) is reflected by the design of the graphical user interface (Figs. 2–5): each tab in the interface represent a milestone in the workflow, and the processes necessary to achieve the respective objectives are bundled in the coloured boxes at the bottom of the panel. The main workflow is grouped into four sections (data processing, initial map calculation, hit identification and refinement) and it is possible to enter at any point, provided that the required data are present in a suitable format.

*XCE* uses a relational SQLite database to store, retrieve and update all of the metadata that are generated as part of a project. The database can be populated by different means depending on the entry point.

(i) In the most general case, *XCE* will be used to process diffraction images, whereby the software will create a sample entry for every data set.
(ii) It is possible to manually create the anticipated directory structure, and the software will be able to auto-populate the database while parsing the project directory. Details of the naming conventions and project directory structure are given in §[Sec sec3.2]3.2 and Supporting Information §S1.
(iii) If the data were collected at the Diamond Light Source then the database can be populated by parsing the respective visit directory as long as the data sets were collected according to the following folder structure: <protein name>/<crystal name>, *e.g.* Lysozyme/Lysozyme-x001 *etc*. All results from automatic data processing are stored in a folder named processed in the respective visit directory, and the folder hierarchy during data collection is reflected in the structure of the processed folder. It contains a directory for each protein target and subfolders for every protein crystal belonging to the target, which then branch into subfolders for different runs and finally into folders for the different data-processing pipelines.
(iv) In the special case where crystals were mounted and collected as part of a crystallographic fragment-screening project at beamline I04-1 at the Diamond Light Source, then the resulting SQLite file can be directly used as input for *XCE*.

*XCE* tracks the status of each mounted crystal by storing the data-collection outcome, refinement stage and ligand confidence in the respective field in the database. An overview and an explanation of the different flags can be found in Supplementary Tables S1–S3. Some of the assignments are performed automatically, while others must be explicitly triggered by the user.

## 3. Software design and implementation   

*XChemExplorer* is implemented in Python (http://www.python.org) and, like *CCP*4, uses the PyQt4 library to provide the graphical user interface functionality. It runs on any Linux or Mac OS X system that has *CCP*4 v.7.0 or higher installed. *XCE* uses SQLite (http://www.sqlite.org) as a relational database-management system. The *RDKit* library (http://www.rdkit.org) is used to create two-dimensional images of compounds. Since *XCE* is a workflow and data-management tool and not an algorithm, it makes use of existing software for different parts of the structure-determination process: *xia*2 (Winter, 2010 ▸) for data processing; *DIMPLE* for initial refinement and map calculation; *AceDRG* (Long *et al.*, 2017 ▸) for generation of ligand coordinates and restraints; *REFMAC* (Murshudov *et al.*, 2011 ▸) for refinement; *PanDDA* (Pearce *et al.*, 2016 ▸) for hit identification; a variety of tools from *PHENIX* (Adams *et al.*, 2010 ▸) for validation purposes; and *Coot* (Emsley *et al.*, 2010 ▸) for model building. There is, however, no fundamental limitation to incorporating other software packages. Source code for *XChemExplorer* and installation instructions are available at http://tkrojer.github.io/XChemExplorer.

### 3.1. Filesystem organization   

*XCE* is a filesystem-based program, meaning that all files are stored in the same project directory and have to be organized in a hierarchical manner. All files belonging to one crystal are stored in a subfolder and the name of this folder is equal to the sample identifier. The filenames have to adhere to a naming convention, but the program will manage the correct labelling if used throughout the process. Nevertheless, the requirements are minimal and it is easy to add data manually by adjusting the folder and file names to the expected nomenclature. All that needs to be provided are an MTZ file and an *AIMLESS* logfile (Evans & Murshudov, 2013 ▸) in the respective sample directory. The files must have the same filename root as the folder name, *e.g.* if the folder name is TEST-x001, then the MTZ and logfile must be called TEST-x001.mtz and TEST-x001.log, respectively. Alternatively, the MTZ file and logfile must have the same name in all subfolders, *e.g.* datafile.mtz and datafile.log. Details of naming conventions are given in Supporting Information §S1.

### 3.2. SQLite database   

*XCE* relies on an SQLite relational database to store and retrieve all of the many pieces of metadata for each sample and experiment. As described in §[Sec sec2]2, the database can be generated in different ways, while its contents can be viewed and edited in the Overview tab (Fig. 2 ▸). An SQLite database is essentially just a file and therefore requires little maintenance, in contrast to more sophisticated server–client database engines which are not easy to set up and maintain without dedicated IT support. *XCE* could be configured to support such systems, but priority was given to establishing a transparent, comprehensible and editable system which reflects the day-to-day reality of the typical practising crystallographer. The database consists of a main table which contains a row for each sample and columns for different metadata, and a secondary table to capture results specific to the novel *PanDDA* (*Pan-Dataset Density Analysis*) hit-analysis software (Pearce *et al.*, 2016 ▸). The location and name of the database is defined in the settings of *XCE* and, since the database is just a file, it can be located anywhere on the filesystem. Access control is file-based, so that any user with read-access to the relevant file system will be able to access the data. The content of the database can be displayed with *XCE* or with freely available software such as the *SQLite Browser* (http://sqlitebrowser.org)

*XCE* can export the database to a comma-separated file (csv), which can then be read into programs such as *Microsoft Excel* for analysis or manipulation. An updated csv file can be reimported into the database if necessary, which is particularly useful for retroactively adding ligand information. *XCE* does not work without a database being specified, but it is always possible to recreate the database from an existing filesystem. In its current form, *XCE* does not provide an interface to external databases, but users who have their own database can easily query the SQLite file and synchronize it with their own system.

### 3.3. Job submission   

*XCE* checks for the availability of a functional queuing system during startup and will submit all computationally intensive calculations to the cluster. Alternatively, the local machine will be used. At the moment only the Portable Batch System (PBS) is supported, but implementation of other queuing systems would not in principle be difficult.

## 4. Description of usage   

### 4.1. Starting the program   

The program can be started with little preparation. It is sufficient to provide a database file and the location of a project directory. If the database does not already exist, then the ‘Data Source’ menu can be used to create a database from scratch. Additional directories can be specified as relevant, for example the location of reference files, the output of *PanDDA* hit analysis or a visit directory at the Diamond Light Source.

### 4.2. Data-collection review and data (re)processing   

The Datasets tab is for reviewing data collections at the Diamond Light Source and for analysing data sets processed with different auto-processing pipelines. Additionally, diffraction images can be (re)processed with the *xia*2 data-reduction system (Winter, 2010 ▸). Historically, this was the first module of the program, because data collected at Diamond Light Source are immediately processed by several automated data-processing pipelines (Winter & McAuley, 2011 ▸). The availability of such a system appears to be indispensable for any ambitious SBLD program since the multiple programs provide redundancy that ensures that the pipeline robustly provides outputs. Individual programs are prone to unpredictable failures in ways that are neither feasible nor interesting to troubleshoot in the face of large amounts of raw data. At the same time, it does present the challenge of having to assess and select one of multiple processed versions of the data for each crystal.

After the processed data are available, the program will walk through the data directories, check for the presence of scaled MTZ files and parse the corresponding *AIMLESS* logfiles. For data collected at Diamond Light Source, it will additionally read and encode crystal images which are recorded before data collection and which indicate whether the specimen was correctly aligned. MTZ and logfiles from all auto-processing pipelines are then immediately copied to the project directory. The program can additionally monitor an ongoing data collection by parsing a specified visit directory every 2 min and dynamically updating the table in the Datasets tab. The current implementation of *XCE* allows only data collected at Diamond Light Source to be reviewed, because the specific directory structure at Diamond is necessarily hard-coded. However, the relevant code is isolated, meaning it can be adapted for other sites with relative ease.

Results are displayed in the central table, showing one line for each sample, together with the currently selected processing results, crystal images after alignment and a drop-down list that specifies the outcome of the experiment (Fig. 3 ▸). *XCE* will copy the final MTZ and logfiles from all pipelines into the project directory, but only the one that is currently selected will be used for any downstream calculations. The program will preselect one result, but it is possible to expand the view and to manually select any data-processing result. Details of the available selection criteria can be found in Supporting Information §S2.

The annotation mechanism is implemented by a drop-down menu for each sample which can be set with a quick scroll of the mouse wheel: this operational simplicity is a vital pre­requisite for data sets to be annotated completely. *XCE* automatically flags data collections as successful if a valid MTZ file is found which fulfils a user-defined resolution criterion. If no MTZ file can be found in a given sample directory then *XCE* will label the data collection as an unknown failure. However, if a user wants to specify the type of failure in more detail, for example no diffraction, loop broken *etc.*, then they can do so by manually changing the text of the drop-down menu (Fig. 3 ▸). Details about the available annotation criteria are given in Supplementary Table S1. The snapshots after crystal alignment are essential for determining the reason for failure: correct crystal alignment is only a peripheral problem for manual data collection, but can be a major issue for automatically centred crystals.

The final facilitator of identifying problematic crystals is the ability to sort the table by any of the displayed categories. Thus, for instance, abnormally large *R*_merge_ values in the inner resolution shell of data sets are usually a good indicator of samples that warrant closer inspection.

### 4.3. Initial map calculation   

The Maps & Restraints panel enables initial refinement of each data set with the *DIMPLE* difference-map pipeline (Fig. 4 ▸). The table provides an overview of all successfully reduced data sets, their resolution and *R*_cryst_/*R*_free_ values in the case where any refinement has been carried out already. *XCE* uses a simple but fast and effective mechanism to search for suitable PDB input files: it will compare the point group and unit-cell volume of each PDB file in the reference directory with the sample MTZ file and suggest it as input for *DIMPLE* if they have the same point group and differ by less than 10% in unit-cell volume. The latter parameter can be adjusted in the Preferences menu. The currently selected reference file is displayed in the drop-down menu, but any of the files in the Reference directory can be chosen. Individual samples or batches can be selected for (re)processing with *DIMPLE*. Since *DIMPLE* is able to solve the structures of non-isomorphous crystals by performing an additional molecular-replacement step, it may not seem necessary to devote much thought to the careful selection of a suitable reference file. However, it is not advisable to completely rely on automated decision making because it is invariably a warning sign if no suitable reference file is available: the program after all assumes that all data sets are similar to the available reference models and it is always better to prepare a good model outside the *XCE* workflow which accurately explains the respective crystal rather than to continue with a suboptimal model. The latter option may sound tempting at the time, but usually results in additional work downstream.

### 4.4. Ligand preparation   

It is a hallmark of SBLD that novel compounds are used that are unlikely to feature in the Cambridge Structural Database or the Protein Data Bank. Hence, ligand coordinates and accurate geometry restraints need to be generated for subsequent model building and refinement purposes (Kleywegt, 2007 ▸). The Maps & Restraints tab allows the calculation of ligand coordinates, restraints and two-dimensional pictures of batches of compounds with *AceDRG* and the *RDkit* library.

Information about the compounds needs to be provided in the SQLite database as a SMILES notation. The resulting files are stored in the respective sample folder in the project directory. The database is updated with information about the location of ligand coordinates and restraints once the files have been successfully created. In case of problems, *XCE* does not analyse the cause for failure, but users can query the database and obtain a quick overview of all failed attempts to generate restraints and find the location of the respective sample folder. Note that ligand coordinates and restraints are automatically loaded into *Coot* during the Refinement stage. Hence, validation and editing functionalities in *Coot* can be used in case the respective values need to be updated (Debreczeni & Emsley, 2012 ▸). Not currently supported are scenarios where the chirality of the compound is unknown, which are not uncommon in ligand-binding studies. In such cases the corresponding files need to be generated manually and copied into the respective folder.

### 4.5. Hit identification (*PanDDA*)   

*XCE* offers a graphical entry point for the recently developed *PanDDA* (*Pan-Dataset Density Analysis*) software (Pearce *et al.*, 2016 ▸) for improved hit identification (Fig. 5 ▸). Interpreting weak and ambiguous ligand density can significantly slow down a project and make the final models less reliable and dependent on subjective decisions of the responsible scientist (Pozharski *et al.*, 2013 ▸), whereas the *PanDDA* algorithm establishes statistically significant measures of confidence for each analysed sample.

The *PanDDA* protocol consists of three steps: analysis, inspection and export. *XCE* displays details of the available data sets for *pandda.analyse* in a table and allows the user to adjust key parameters of the analysis protocol. The results can be displayed with *pandda.inspect*, which uses *Coot* and a special inspection panel for assessing and modelling of the bound ligands. The resulting models undergo a round of refinement and validation during the export step. Additionally, all output which is generated by the *PanDDA* software will be imported into the database at this point. Details of the processes taking place during the export step are given in Supplementary Fig. S2. While it is recommended to use *PanDDA* for hit identification, it is not necessary to be able to continue with the workflow since it is possible to proceed directly to refinement after initial map calculation.

### 4.6. Refinement   

Completing structures requires iterative cycles of model building, refinement and validation, with progress monitored by the convergence of global quality metrics. Unfortunately, the endpoint is often ill-defined and mostly depends on the assessment of a skilled crystallographer. *XCE* addresses this challenge by minimizing the number of cycles through assisting the user in obtaining the best possible starting model (see §§[Sec sec4.3]4.3 and [Sec sec4.5]4.5).

**Figure 6:**
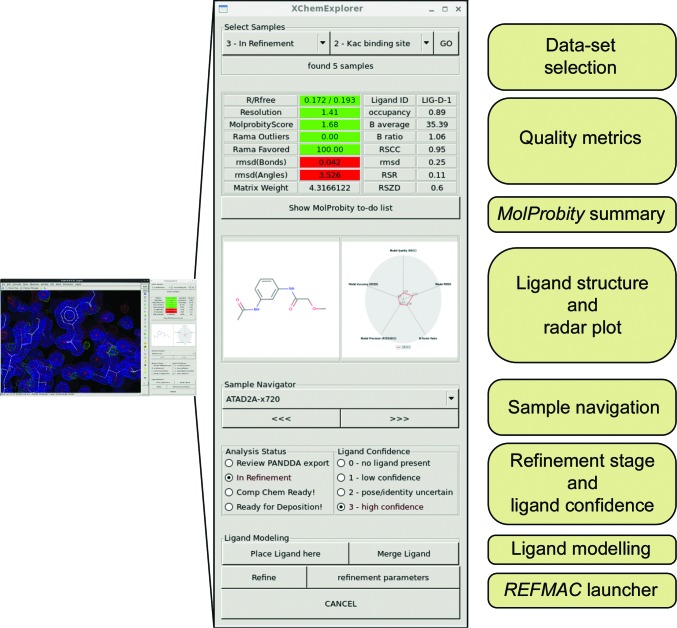
The *XCE* Refinement interface for accelerated model building and refinement. The small panel on the left side shows how *Coot* and the new dialogue work in relation to each other; the middle and right side show an extended image of the dialogue and a schematic outline of its arrangement. The dialogue displays global and ligand-specific quality metrics of the currently loaded structure as well as a two-dimensional picture of the ligand and a radar plot summarizing a set of ligand-validation scores. *XCE* will automatically display 2*mF*_o_ − *DF*_c_ and *mF*_o_ − *DF*_c_ maps as well as *PanDDA* event maps when they are available. The arrow buttons and a drop-down menu in the control panel are used to step through the data sets. It also allows the refinement stage of a given structure and the confidence of the modelled ligand to be assigned. All structures which are part of the project can be handled within a single instance of *Coot*.

The process is greatly accelerated by a graphical command interface which allows modelling and refinement of all data sets within a single instance of *Coot* (Fig. 6 ▸). A subset of all available structures can be selected based on their refinement stage and, in the case where the ligands were built with *PanDDA*, location of the respective binding site. The interface displays global and ligand-specific quality metrics of the currently loaded structure, as well as a two-dimensional picture of the ligand and a radar plot summarizing a set of ligand-validation scores: correlation between model and observed electron density (RSCC), a statistical measure of difference density in this region of the model (RSZD), the *B*-factor ratio of ligand and surrounding protein side-chain atoms within 4 Å (*B*-factor ratio), the root-mean-squared deviation of ligand coordinates in the initial model and after refinement (r.m.s.d.), and the strength of density over model normalized for occupancy (RSZO/OCC) (Pearce & von Delft, 2017 ▸; Tickle, 2012 ▸). The corresponding ligand coordinates and restraints are loaded as a separate molecule in C*oot* when a new molecule is chosen. In the case where the ligand had not been modelled before, then the interface can be used to first place the molecule into the centre of the screen for further fitting and then to merge it into the loaded protein structure. Refinement can be triggered with a single button click and is carried out with *REFMAC*, followed by comprehensive valid­ation with different programs from the *PHENIX* package. Details of the procedure can be found in Supplementary Fig. S3. Finally, global and ligand-specific quality metrics of the resulting model are determined and the database is updated accordingly.

Additionally, a qualitative assessment of refinement stage and ligand confidence of each sample can be chosen from pre-defined categories. *XCE* uses a hierarchical annotation system to help the refiner describe the refinement stage of a given data set, facilitating the organization and prioritization of samples within a project. The respective database field is updated automatically during data collection, initial refinement with *DIMPLE* and *PanDDA* analysis, but once the final refinement stage is reached crystallographers need to change the flag individually. The categories are mostly self-descriptive and details can be found in Supplementary Table S2. Introduction of this hierarchy helps to provide an overview of the work that is still needed to finish all the models, and it allows subsets of all available structures to be selected which need more work to be performed before they are ready for subsequent analysis and deposition. The ligand confidence category reflects the trust of the refiner in the modelled ligand. This may change as refinement progresses and is informed by how well the electron density or the *PanDDA* event map describes the ligand and by the quantitative metrics described above. Ultimately, it remains a subjective statement, but it is especially useful in cases where the crystallographer cannot unambiguously establish the binding pose and therefore needs a way to communicate the limitations of the model to whoever uses it for further analysis.

### 4.7. Data sharing   

The final problem addressed by *XCE* is how to communicate the results with other scientists; this is of particular importance in SBLD projects, which are per definition multi-disciplinary. The software tools and quality metrics that are well known to the crystallographic community tend to be alien to non-structural biologists, and not in fact relevant to their problems. In our experience, they ask the following questions.

(i) Which protein–ligand structures are available?
(ii) Where is the PDB file?
(iii) Is this a ‘good model’?
(iv) Is that water/methyl/hydroxyl really where it was modelled?

**Figure 7:**
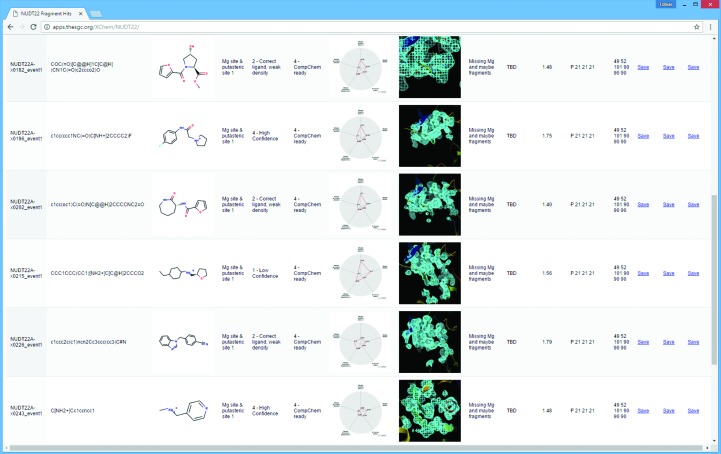
Data sharing *via* HTML export. Results generated with *XCE* can be exported as an HTML document, which can be put on a web server and shared with other scientists. The figure shows the contents of the summary page; details of each modelled ligand and download links are displayed on one line. Additionally, an interactive *IcmJS* plugin (Molsoft LLC) allows display of the ligand-binding site and the respective *PanDDA* event map.

*XCE* therefore includes a tool which converts the results in the database into an HTML document (Fig. 7 ▸). The HTML format allows the easy sharing of results either within an organization or with other scientists *via* the internet. The bound ligands are displayed in a tabular form, including details of the compound, manual annotations of reliability and links to download PDB, MTZ and CIF files. Most importantly, the recipients can themselves assess the reliability of the model: numerically by a radar plot that summarizes the reliability of the ligand and model (Pearce & von Delft, 2017 ▸) and graphically through an interactive *IcmJS* plugin (Molsoft LLC) that displays the primary evidence for the ligand, namely the *PanDDA* event map.

## 5. Results and discussion   

*XCE* has been extensively tested by supporting crystallo­graphic fragment screening at Diamond Light Source. In the course of several iterations of bug fixes, the program has become one of the cornerstones of the facility: only once the program was available in its current stable form did it become possible to completely model fragment-screening projects promptly and efficiently.

The philosophy of the program was stress-tested in a retrospective analysis of six historic fragment campaigns where the goal was to detect and model all bound ligands with the novel *PanDDA* algorithm and assemble information about how the fragment set influences the diffraction behaviour of different crystal systems. The review of 5970 individual data sets was completed by two experienced crystallographers in only 5 d, including the documenting of all experiments and finalizing all 380 protein–ligand structures. This was only possible because the program condensed huge amounts of information to make it comprehensible for the user. For instance, many data-quality statistics are available, but we confirmed that two criteria, namely the resolution limit, defined as where Mn〈*I*/σ(*I*)〉 falls below 1.5, and the *R*_merge_ value for the inner resolution shell, were wholly sufficient for identifying problematic data sets. Additionally, the representation of results in tables and the possibility to sort them made it straightforward to triage data sets into those that could be taken forward and those that needed further attention. Therefore, most of the work up to manual inspection of the *PanDDA* output was completed with minimal user input. Here too, only around 20% of the all data sets had to be checked for bound ligands, as they were the only ones with a statistically significant difference signal. Finally, all steps that required manipulation of models or decision making by the refiner were facilitated because all commands and annotations could be carried out within a single instance of *Coot*, allowing the users to finish tens of structures within a few hours.

While *XCE* implements all steps required to solve protein–ligand structures, the program is designed to accommodate multiple entry points, since different projects have different requirements and not all will always conform to the standard workflow. For example, while the *PanDDA* algorithm is known to be a powerful tool for hit identification, if a user decides that this is not applicable, *XCE* nevertheless supports inspection of initial electron-density maps, ligand building and refinement.

*XCE* depends on a relational SQLite database, but is at its heart a filesystem-based tool. While a server-based database system may have certain advantages and seem more elegant, the chosen paradigm is more transparent for the responsible scientists and allows the introduction of changes without the user having to be familiar with relational database systems. However, there are clearly some limitations to this approach, which other recently presented developments have circumvented, including *ManageCCP*4*i*2*Archive*, a tool for the collaborative sharing of large sets of structural projects undertaken across a research group, written in the *CCP*4*i*2 structure-determination environment but using a web-based approach (M. Noble, personal communication). Both developments clearly point in the same direction and could indeed be combined with relative ease for multi-disciplinary research projects: the structures and results generated by *XCE* could be fed into *ManageCCP*4*i*2*Archive*, which could then be used to distribute the information to all the scientists involved in the project, providing a high-level platform for further structural analysis.

The algorithmic simplicity of the workflow means that most apparent errors are in fact caused by undetected crystallo­graphic problems. The unexpected appearance of new crystal forms for a subset of the collected crystals is a typical case where the underlying problem can be quickly and easily established with *XCE*. In particular, discrepancies between the reference files provided and the data sets will almost always show up in the Maps & Restraints table when the point groups are not in agreement, the differences between unit-cell volumes are large or the *R*_cryst_/*R*_free_ values are high after initial refinement. The sorting mechanism allows the identity and the number of data sets affected to be rapidly identified. Additionally, more subtle differences between the data sets can be detected with a *PanDDA* data-set clustering algorithm.

Although deposition procedures at the Protein Data Bank are well established, using them to deposit dozens of structures is not realistic without helper tools. The PDB is currently developing a bulk deposition interface to encourage groups which are working on SBLD projects to contribute their structures (Stephen K. Burley, personal communication). Even so, a major bottleneck remains that all the required metadata must be collated; as *XCE* already records much of the required information, it can automatically generate valid mmCIF files. This required functionality will be fully implemented in the near future.

## Supplementary Material

Supporting Information.. DOI: 10.1107/S2059798316020234/ba5267sup1.pdf
