# Supplementary material for: The XChemExplorer graphical workflow tool for routine or large-scale protein–ligand structure determination
Source: Acta Crystallogr D Struct Biol. 2017 Feb 24;73(Pt 3):267–78. doi: 10.1107/S2059798316020234 (PMC5349439; doi:10.1107/S2059798316020234)
Supplement: Supplementary file 1 [file d-73-00267-sup1.pdf]

STRUCTURAL BIOLOGY

**Volume 73 (2017)**

**Supporting information for article:**

## The *XChemExplorer* graphical workflow tool for routine or large-scale protein–ligand structure determination

**Tobias Krojer, Romain Talon, Nicholas Pearce, Patrick Collins, Alice Douangamath, Jose Brandao-Neto, Alexandre Dias, Brian Marsden and Frank von Delft**

### S1. Project directory structure and file name conventions

The project directory structure of XChemExplorer is as follows:

`<project_directory>/<sample_id>`

e.g.

`/Users/tobiaskrojer/SGC/PHIPA/fragment_screen/PHIPA-x001`

`/Users/tobiaskrojer/SGC/PHIPA/fragment_screen/PHIPA-x002`

`/Users/tobiaskrojer/SGC/PHIPA/fragment_screen/PHIPA-x003`

etc.

Each sample folder contains a *MTZ* file and the corresponding *AIMLESS* logfile, e.g.:

`PHIPA-x001.mtz`

`PHIPA-x001.log`

XCE uses a file called `<sample_id>.free.mtz` as input for refinement. This file is either automatically generated by the DIMPLE difference map pipeline or users can choose to append an existing Rfree set from a reference file by providing it in the reference folder.

*MTZ* column labels must have *CCP4* default names, otherwise XCE may show unexpected behaviour, i.e. IMEAN, SIGIMEAN, F, SIGF, FreeR_flag. The program can only parse *AIMLESS* logfiles at the moment.

After *DIMPLE* is run successfully, the resulting *PDB* and *MTZ* files will be linked as `dimple.pdb` and `dimple.mtz` into the respective sample directory.

Once the refinement stage is reached a subfolder for each refinement cycle will be created: `Refine_<cycle number>`. This subfolder contains the modified *PDB* file, executable shell script and output. The script contains the complete refinement and validation schedule. After successful refinement, the resulting *PBD* and *MTZ* files will be linked as `refine.pdb` and `refine.mtz` into the sample directory.

It is possible to create this folder structure manually and then choose *Data Source* -> *Update Data Source from filesystem* from the *XCE* menu to import all the information into the database.

### S2. Dataset selection of different auto-processing pipelines

It is difficult to know what the best output is when data were processed with different settings and the resulting data collection statistics appear similar. *XCE* offers a default selection mechanism which will be described below but also offers selection by obvious criteria like highest resolution.

Crystal systems used for SBLD are often well characterised, hence the default selection mechanism tries to pick only data processing result which have the same point group and a similar unit cell volume as one of the provided reference *PDB* files. It tries to eliminate datasets with suspiciously high low resolution $R_{merge}$ values and assigns an empirical score to each outcome which serves as the final discriminator. The score is defined as:

$$score = \frac{N(reflections) \times Completeness \ \times Mn\left(\frac{I}{sig(I)}\right) \times N(ASU)}{unit\ cell\ volume}$$

Where N(reflections) is the number of unique reflection, Completeness is the overall completeness, Mn(I/sig(I)) is the overall signal-to-noise ratio, N(ASU) is the number of asymmetric units per unit cell for a given point group. Details of the selection mechanism are given figure S1.

**Table S1** The following pre-defined categories are used to annotate the overall data collection outcome.

Some of the categories are only relevant when data were collected in automatic mode.

| | |
|---|---|
| success | Data collection was successful. |
| centring failed | The crystal was not correctly centred in one or several orientations. |
| no diffraction | The crystal was correctly centred, but does not diffract. |
| Processing failed | The crystal showed satisfactory diffraction, but none of the data processing pipelines was able to process it automatically. |
| loop empty | No crystal was present in the loop. |
| loop broken | The loop of the sample holder containing the crystal fell off. |
| low resolution | Automated data processing was successful, but the maximum resolution is lower than the user deems it acceptable; the value can be adjusted in the Preference menu (default is 3.5Å). |
| no X-rays | Diffraction images are blank; a dataset was collected without exposure of the crystal to X-rays. |
| unknown | This is the default setting if diffraction images were found, but no corresponding processing results seem available. XCE does not attempt to determine the cause of failure. |

**Table S2** The table summarises the categories which are used to describe the refinement stage of a given dataset.

| | |
|---|---|
| -2 – Refinement failed | Refinement failed |
| -1 - Data collection failed | Data collection failed; details of which are found in the data collection outcome field of the respective sample. |
| 0 – Dataset collected | A datasets was successfully collected, but no further actions were initiated, yet. |
| 1 - Analysis pending | Initial maps were calculated, but not analysed. |
| 2 - PANDDA model | A protein-ligand structure has been built with pandda.inspect but not refined, yet. |
| 3 - In Refinement | The dataset is currently being refined. |
| 4 - CompChem ready | The structure is ready for analysis because all regions of interest, e.g. the ligand binding site, are modelled with confidence and the overall quality indicators are satisfactory. There may still be local errors in other parts of the model. |
| 5 - Deposition ready | The model is ready for deposition into the Protein Data Bank |
| 6 – in PDB | The structure has been deposited into the Protein Data Bank. |

**Table S3** Ligand confidence categories

The categories below are used to qualitatively characterize the confidence the refiner has in the modelled ligand. Low, weak and unexpected ligands will often not be suitable for deposition into the PDB, but this information can be useful for analyzing the project as a whole.

| | |
|---|---|
| 0 - no ligand present | No ligand has been built, because neither 2mFo-DFc, mFo-DFc or *PanDDA* event maps support the presence of a bound ligand. |
| 1 - Low Confidence | A ligand has been built, but the confidence of the refiner in its pose and identity is low. These models need to be treated with utmost suspicion and should only be looked at in connection with the respective 2mFo-DFc, mFo-DFc or *PanDDA* event maps. |
| 2 - Correct ligand, weak density | The respective 2mFo-DFc, mFo-DFc or *PanDDA* event maps basically agree with the specified ligand, however, the signal remains ambiguous. Hence, the pose of the ligand could not be established with certainty and the model needs to be treated with scepticism. |
| 3 - Clear density, unexpected ligand | The respective 2mFo-DFc, mFo-DFc or *PanDDA* event maps are very well defined, but their shape does not agree with the specified ligand. It is recommended to the check if a mix-up has happened or if the chemical composition of the sample is as stated. The experiment should be repeated. |
| 4 - High Confidence | The respective 2mFo-DFc, mFo-DFc or *PanDDA* event maps are very well defined and agree with the specified ligand. The ligand could be modelled with high confidence. |

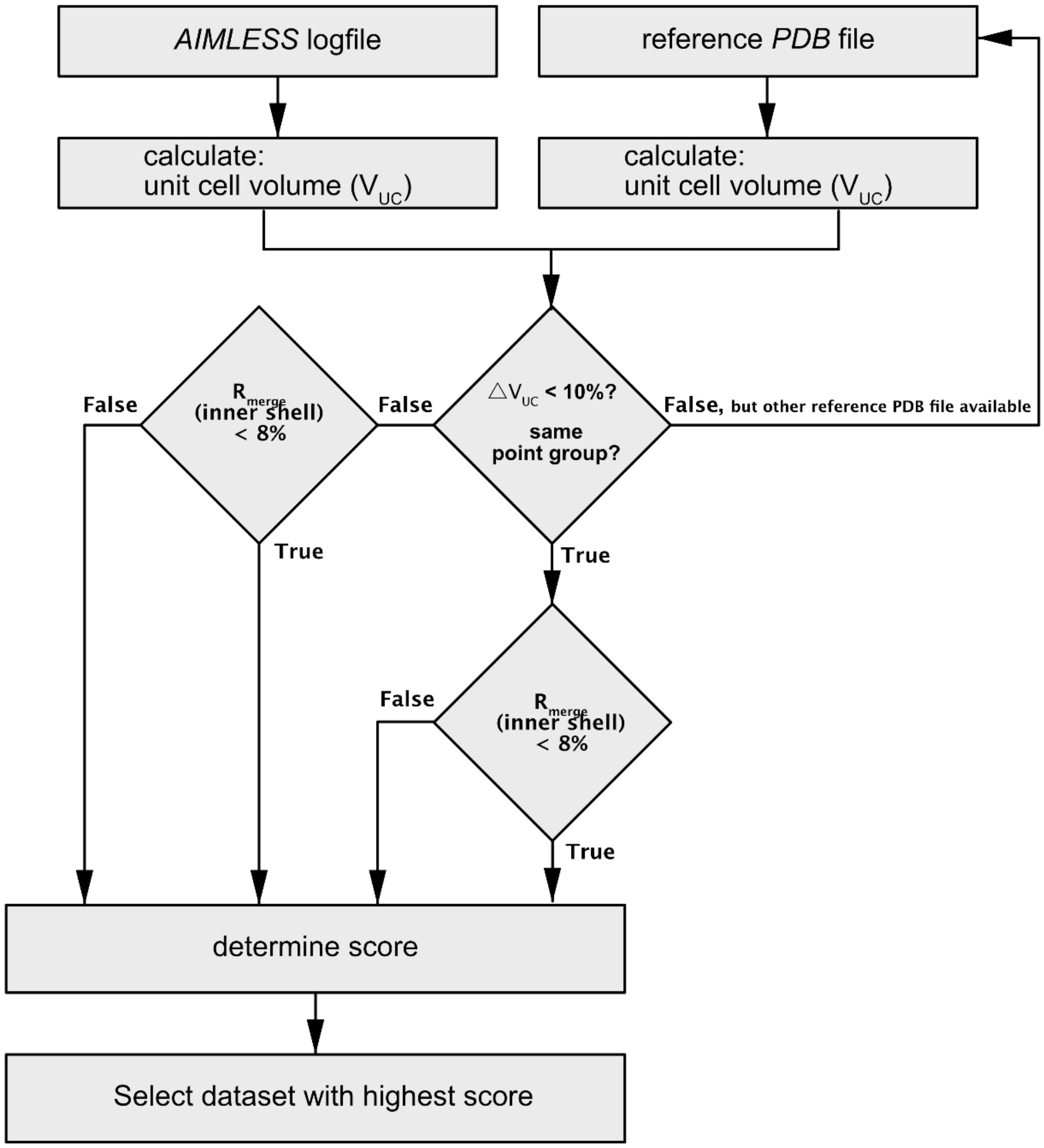


**Figure S1** A schematic of the default auto-processing selection mechanism. Initially, the program iterates until it finds a reference file which has the same point group and similar unit cell volume as the analysed dataset. All datasets which satisfy a certain stage are taken forward. Finally, an empiric score is determined and the dataset with the highest score is selected.

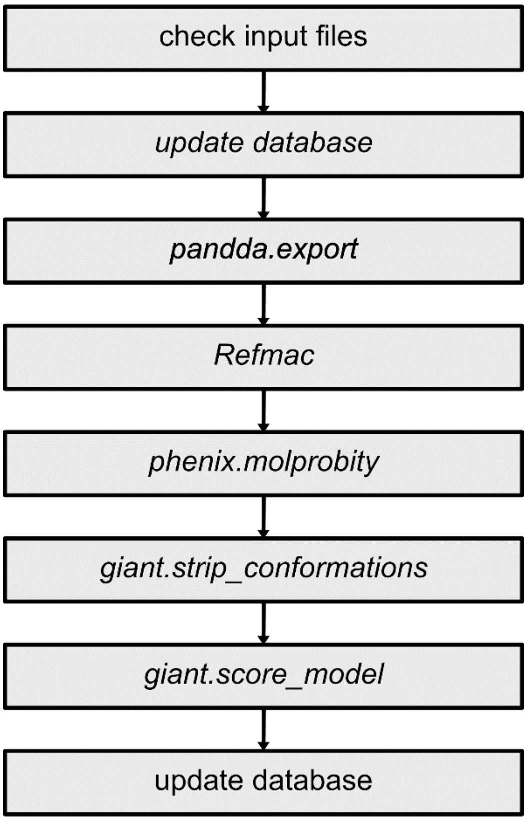


**Figure S2** A schematic of the *PanDDA* export procedure. The flowchart illustrates the different steps which are triggered when models built with *pandda.inspect* are exported back to the crystallographic unit cell. The actual back transformation of coordinates and event maps is done by *pandda.export*; *giant.strip_conformations* prepares a structure devoid of any conformations representing the unbound state of the protein and *giant.score_model* calculates a series of scores for each modelled ligand. The afore mentioned programs are all part of the *PanDDA* software suite.

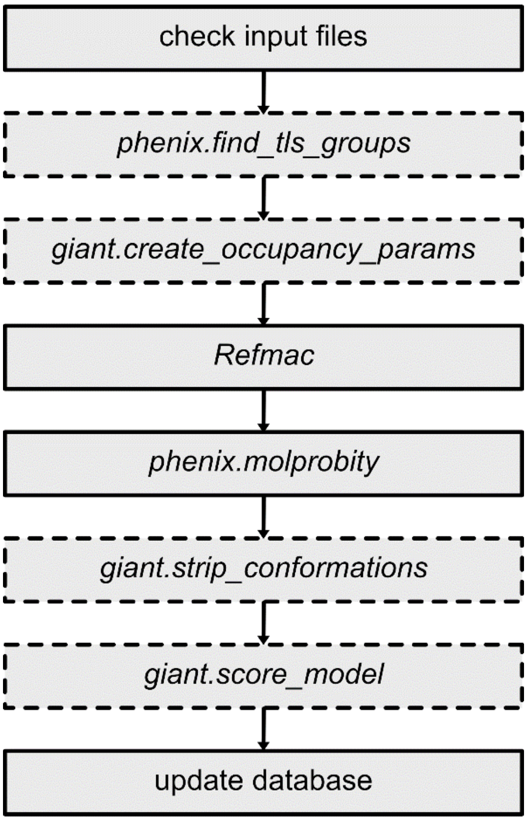


**Figure S3** A schematic of the refinement protocol. Optional modules which are either chosen by the user (determination of TLS groups) or which are only relevant when structures where modelled with *pandda.inspect* are highlighted with dashed borders.
